# GPS Spoofing Detection Method for Small UAVs Using 1D Convolution Neural Network

**DOI:** 10.3390/s22239412

**Published:** 2022-12-02

**Authors:** Young-Hwa Sung, Soo-Jae Park, Dong-Yeon Kim, Sungho Kim

**Affiliations:** Agency for Defense Development, Yuseong P.O. Box 35, Daejeon 34186, Republic of Korea

**Keywords:** GPS spoofing attack, small UAV, deep learning, 1D CNN, SVM

## Abstract

The navigation of small unmanned aerial vehicles (UAVs), such as quadcopters, significantly relies on the global positioning system (GPS); however, UAVs are vulnerable to GPS spoofing attacks. GPS spoofing is an attempt to manipulate a GPS receiver by broadcasting manipulated signals. A commercial GPS simulator can cause a GPS-guided drone to deviate from its intended course by transmitting counterfeit GPS signals. Therefore, an anti-spoofing technique is essential to ensure the operational safety of UAVs. Various methods have been introduced to detect GPS spoofing; however, most methods require additional hardware. This may not be appropriate for small UAVs with limited capacity. This study proposes a deep learning-based anti-spoofing method equipped with 1D convolutional neural network. The proposed method is lightweight and power-efficient, enabling real-time detection on mobile platforms. Furthermore, the performance of our approach can be enhanced by increasing training data and adjusting the network architecture. We evaluated our algorithm on the embedded board of a drone in terms of power consumption and inference time. Compared to the support vector machine, the proposed method showed better performance in terms of precision, recall, and F-1 score. Flight test demonstrated our algorithm could successfully detect GPS spoofing attacks.

## 1. Introduction

Small unmanned aerial vehicles (UAVs) of approximately 30 kg, represented by quadcopters, have been widely used in civil and military fields, and their practical use is rapidly increasing. Because the global positioning system (GPS) sensor is comparatively precise among the many sensors in small UAVs, their positioning and navigation are highly reliant on GPS.

In recent years, the vulnerability of small UAVs to GPS spoofing attacks has been studied. Kerns et al. succeeded in a GPS spoofing attack on a helicopter [1]. Noh et al. proposed a method to hijack drones using a GPS spoofing technique called meaconing [2]. Numerous GPS spoofing detection methods have been introduced [3]. However, they are unsuitable for small UAVs because their capacity (e.g., space, battery, and payload) is not affordable to add heavy hardware [4].

Recent advances in deep neural network (DNN) provide a valuable tool to detect anomalies in time-series data [5]. A one-dimensional (1D) convolutional neural network (CNN) is suitable for mobile devices that require real-time operability owing to its low-cost implementation [6].

This study presents a GPS spoofing detection method based on 1D CNN. Our method can detect a GPS spoofing attack before GPS position falsification, contrary to previous study [7]. In addition, the adopted model is lightweight and can be executed on an embedded board (e.g., NVIDIA Jetson Nano or Xavier) of small UAVs. Furthermore, we evaluated the power consumption and inference time of our model on corresponding boards. Finally, the developed algorithm was employed on the Pixahawk drone, and its feasibility was demonstrated in flight test. The contributions of our work are summarized as follows:

- To our knowledge, this study is the first to deploy a deep learning-based spoofing detection model in drone and validates the model in flight test. Most spoofing detection studies have conducted in simulation environments.
- Previous researches fall short of addressing the defense against an intermediate spoofing attack in real test. However, using 1D CNN model, we could circumvent the attack, whereas commercial drones became out of control in the field test.
- Inference time and power consumption are the important aspects for mobile platform applications. So far, most of related works are only focused on performance of machine learning model. We evaluated them and showed that our proposed method was fitted for the operation of small UAVs.

The remainder of this paper is organized as follows. GPS spoofing detection methods applicable to small UAVs are summarized in Section 2. In Section 3, we suggest a GPS spoofing detection method based on 1D CNN accommodating residual network (ResNet). The inference time and power consumption of 1D CNN on an embedded board are evaluated in Section 4. Before flight test, we tested the efficiency of the spoofer for DJI Phantom 4 and Mavic. We modified the Pixahawk drone and equipped it with 1D CNN. The anti-spoofing enabled drone successfully detected a spoofing attack and safely returned to the base during the flight test. Further research directions and conclusions are presented in the end of Section 4 and Section 5.

## 2. Related Works

### 2.1. Taxonomy of GPS Spoofing Signals and Attacks

#### 2.1.1. Types of GPS Spoofing Signals

As listed in Table 1, GPS spoofing signals can be classified into meaconing and generative spoofing.

Meaconing signal is generated by recording and rebroadcasting an authentic GPS signal. Therefore, the signal has a time offset from that of authentic GPS. In addition, a UAV can be spoofed if the target receiver is in GPS signal acquisition mode. If it reaches signal-tracking mode, a meaconing attack requires jamming for the target receiver to re-acquire GPS signals [2].

Generative spoofing requires a subtler approach. It can synchronize GPS time and modulate navigation messages using a spoofing simulator. To capture the tracking loops of the target receiver, the signal is emitted at low power, and its power is gradually increased. Subsequently, the victim receiver is dragged to a counterfeit position. Snapshots of the steps enabling spoofing attacks are illustrated in [3]. Detailed requirements for successful spoofing attacks are described in [8]. Because a generative spoofing attack maintains a lock during the process, detection can be avoided even when the target receiver is in GPS tracking mode. Because meaconing signal can be easily discriminated by checking the time offset, we suggest that generative spoofing is a foreseeable threat for small UAVs. The following section describes the GPS spoofing types with respect to sophistication of the attacks.

#### 2.1.2. Classification of GPS Spoofing Attacks

GPS spoofing attacks are further classified into three levels of difficulty: simplistic, intermediate, and sophisticated [9,10], as listed in Table 2. A simplistic attack utilizes GPS simulation software, such as software-defined radio. It uses a low-cost GPS signal simulator but does not offer time synchronization. Therefore, GPS spoofing is possible only when the target receiver is in signal acquisition mode. Otherwise, jamming should be accompanied by the target receiver to locate other GPS signals.

The intermediate attack via a portable receiver spoofer synchronizes its signal to GPS time, and it can generate false signals that align with the authentic signals. Compared with the simplistic approach, this synchronized attack is relatively expensive and requires additional clock-generating hardware and software. This type of attack is likely to be a significant risk to UAVs in the future because it can manipulate the target receiver in the signal-tracking mode.

A sophisticated attack is coordinated using multiple phase-locked spoofers. It can thwart the target receiver with an angle-of-arrival defense. According to [8], cryptographic authentication is the only known countermeasure. However, this attack is not feasible to spoof small UAVs because the system setup is complex and expensive. Among three attacks, we consider the intermediate attack to be the most significant near-term threat to small UAVs. The following discussion focuses on the detection and prevention of intermediate attacks.

### 2.2. GPS Spoofing Detection Techniques

Hardware-based GPS spoofing detection methods have been introduced, such as phased-array antennas, multiple receivers, attitude and heading reference system (AHRS)/accelerometer and cryptographic systems [11,12]. However, these methods cannot be employed because of the hardware capability (e.g., space and battery) of small UAVs. Recent studies have focused on data-driven and software-based GPS spoofing detections. Various machine learning approaches were exploited in global navigation satellite system (GNSS) use cases, including DNN [13,14,15,16,17,18,19,20,21,22]. However, the performance of the previous approaches was tested in simulation environments. Most studies have not validated their efficiency in real-world environments, such as drone flight tests.

We narrowed the candidates of applicable methods to small UAVs down to four categories as follows [4,11,23]:

- M1: Consistency check of data within the GPS PVT (Position, Velocity, Time) solution
- M2: Monitor the relative GPS signal strength
- M3: Monitor the signal strength of each received satellite signal
- M4: Monitor space vehicle identification codes and number of received signals

Before moving on to introduce our approach, the stages of the intermediate spoofing attack should be addressed. It is divided into a signal hijacking stage and a position falsification stage [8]. In the hijacking stage, the target receiver is forced to capture the signal which has a stronger power with no time offset between the generated one and authentic GPS signal. After pseudo-position information is inserted into the generated signal, the position of target UAV is falsified.

The consistency check of an inertial navigation system (INS) and GPS was proposed for detecting GPS spoofing [7]. In our opinion, this approach is inadequate because it is the post-detection of the spoofing attack in the phase of position falsification. It is impossible to prevent the signal hijacking. Therefore, M1 is not a suitable countermeasure for our purpose. We eliminated M4 in our candidates, because it cannot utilize pattern analysis of time-series data.

We chose the method, M2, which monitor the average signal strength of all satellites, and M3, which compare the signal strength of between satellites. We also used the GPS service Daemon (GPSd) to collect GPS data [24] and acquired the TPV and SKY class data using the NMEA-0183 interface, as shown in Table 3. Integrating the above-mentioned approach and GPSd, we try to detect deception in the hijacking signal stage prior to position falsification.

First, we extracted three features: the mean (snr_mean), difference (snr_range1), and standard deviation (snr_range2) of received satellites’ signal to noise ratios (SNRs) in SKY class. Second, we preprocessed the acquired GPS data as follows:

- snr_mean = mean (snr);
- snr_range1 = max (snr) − min (snr);
- snr_range2 = standard deviation (snr);
- where snr is given by the list (SNRs of GPS satellites used in fix).

## 3. GPS Spoofing Detection Method Based on Deep Learning

### 3.1. One-dimensional CNN Model

A CNN model was developed for image recognition [25]. By replacing 2D image data with time-series data, the CNN model can also be applied to detect anomalies in time-series data [26].

**Figure 1:**
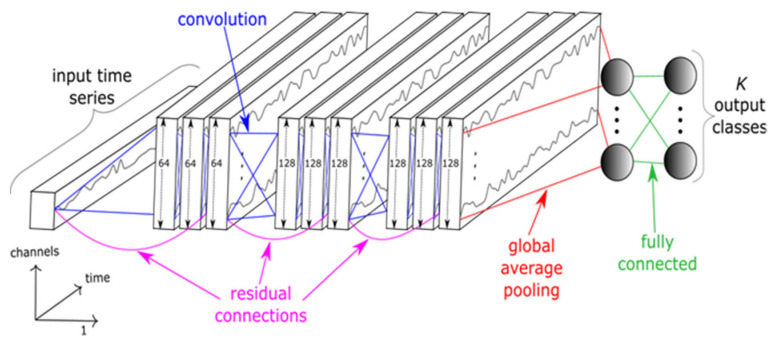
One-dimensional CNN (ResNet) structure [26].

Because the 1D CNN performs scalar multiplications and additions, its computational complexity is significantly lower than that of 2D CNN. It has a competitive advantage where training data is scarce and fast inference is required. Thus, it is a viable option for mobile platform applications because of its real-time operation and low-cost hardware implementation [6]. We adopted the ResNet architecture and its structure is shown in Figure 1 [26,27].

ResNet consists of nine convolutional layers followed by a global average polling layer and a fully connected layer. In ResNet, adding the shortcut residual connections in each residual block enables an efficient training of DNNs by mitigating vanishing gradient problem. Each residual block consists of 3 convolution layers and their ouput is added to the input of residual block. Then, it is fed to the consecutive layer [26]. After each convolution, batch normalization and rectified linear unit activation function are used.

Convolutional layers generate a number of feature maps of 64, 128, and 128, respectively. A global average pooling layer reduces the dimensionality and converts the data into 1D array. We used sigmoid function in a fully connected layer for binary classification (Authentic/Spoofing).

### 3.2. Data Preprocessing

**Figure 2:**
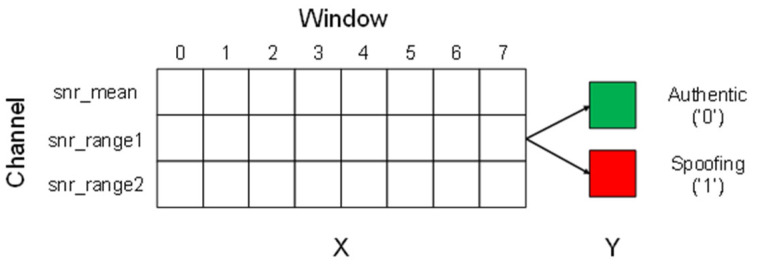
Data preprocessing procedure.

Data preprocessing procedure is as follows. We selected three features, snr_mean, snr_range1, and snr_range2, as shown in Figure 2. The training data were preprocessed by a 3 × 8 matrix with a window size of three channel data and eight ticks. Each vector of the aforementioned matrix was labeled such that authentic signal was “0” and spoofing signal was “1”.

**Figure 3:**
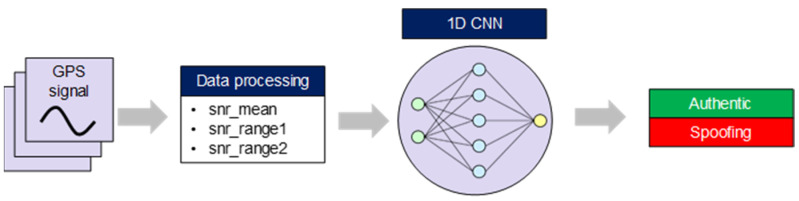
Spoofing detection procedure by deep learning approach.

After training with the labeled data, GPS data is discriminated as ”0” (authentic signal) or “1” (spoofing signal) using the 1D CNN inference model (Figure 3).

To determine the size of slicing window, we examined precision, recall, and F-1 score with respect to different window sizes from 4 to 20. They are the performance metric of evaluating machine learning algorithms and calculated as follows:

(1) $$\mathrm{Precision}=\frac{\mathrm{True}\;\mathrm{Positive}\;\left(\mathrm{TP}\right)}{\mathrm{True}\;\mathrm{Positive}\;\left(\mathrm{TP}\right)+\mathrm{False}\;\mathrm{Positive}\;\left(\mathrm{FP}\right)}$$

(2) $$\mathrm{Recall}=\frac{\mathrm{True}\;\mathrm{Positive}\;\left(\mathrm{TP}\right)}{\mathrm{True}\;\mathrm{Positive}\;\left(\mathrm{TP}\right)+\mathrm{False}\;\mathrm{Negative}\;\left(\mathrm{FN}\right)}$$

(3) $$F\text{-}1\;\mathrm{score}=\frac{2}{\frac{1}{\mathrm{precision}}+\frac{1}{\mathrm{recall}}}$$

where TP, TN, FP, and FN are the components of confusion matrix in Table 4.

**Figure 4:**
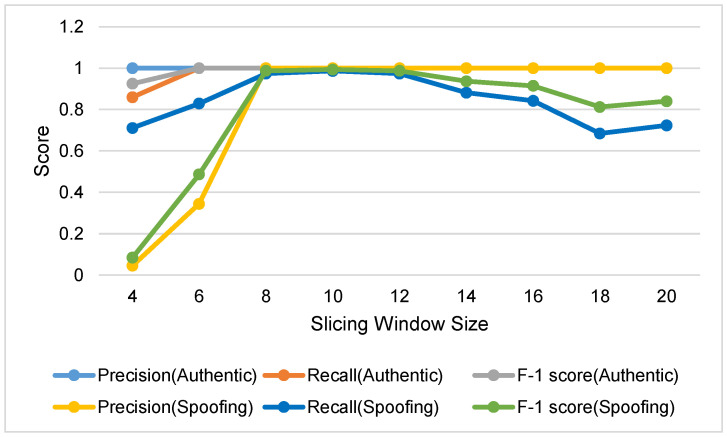
Precision, recall, and F-1 score with respect to slicing window size.

As shown in Figure 4, for authentic GPS signals, most scores in all cases are close to 1 when the window size is greater than 6. It is seen that optimal range of window size lies within 8 to 12. The scores of window size of 8 are not only comparable to those of window size of 10 or 12, but it can also achieve faster response. Consequently, we determined the window size as 8.

## 4. Experiments

### 4.1. Environment to Simulate the GPS Spoofing Signal

**Figure 5:**
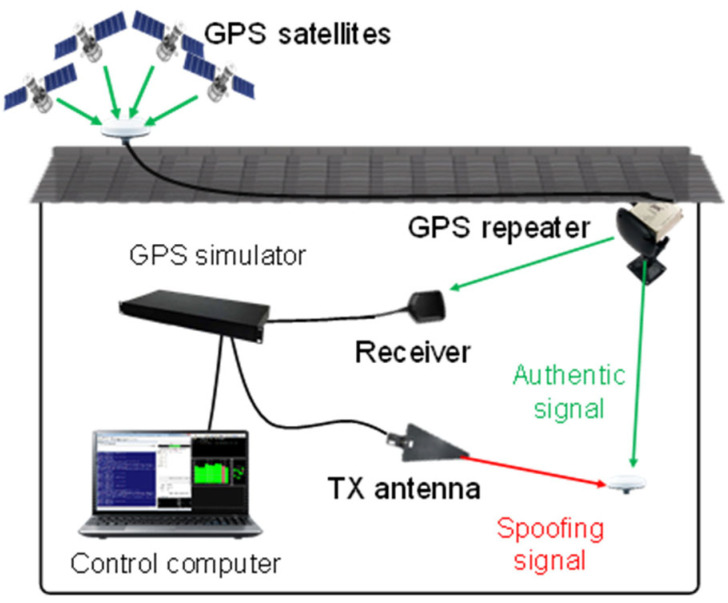
GPS spoofing signal simulation environment.

To acquire sufficient data, we set up an environment to simulate GPS spoofing signals, as shown in Figure 5.

GPS signal was relayed using both an exterior antenna and a GPS repeater. Spoofing signal was generated in a spoofing simulator and entered a GPS receiver through a TX antenna. Thus, we acquired both authentic and spoofing GPS signals simultaneously.

**Figure 6:**
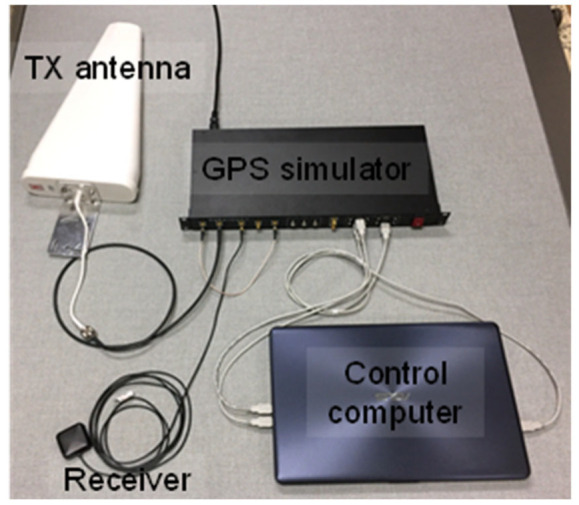
Configuration of GPS spoofer.

We utilized the receiver spoofer that can emit intermediate spoofing signals. The receiver spoofer comprises a GPS spoofing simulator, TX antenna, receiver, and control computer, as shown in Figure 6. It performs the following function: receives GPS satellite signals, generates clocks/spoofing signals, and amplifies them. We also developed an in-house program to monitor the current GPS signal status and control the spoofing signals. Controllable factors include amplitude of the generated spoofing signals, time offset, and position falsification information.

### 4.2. Spoofing Data Analysis

**Figure 7:**
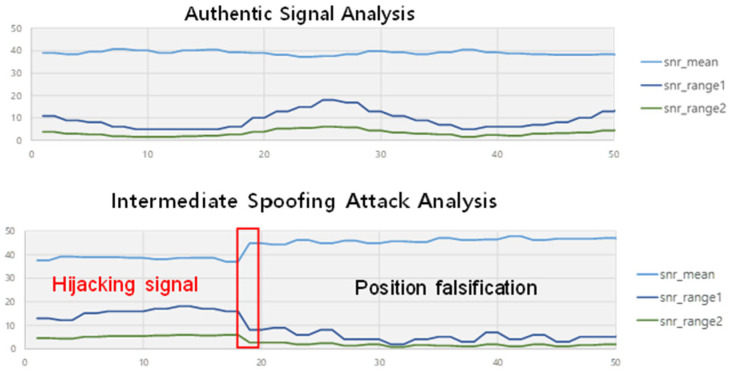
Spoofing data analysis.

According to [8], GPS signal can be stably snatched when the relative difference in the signal strength between spoofer and receiver exceeds 2 dB. In this study, we acquired spoofing data in which the relative difference of the signal strength ranged from 2 to 8 dB. The spoofing signal data are shown in Figure 7.

It is observed that snr_mean increases; however, both snr_range1 and snr_range2 decrease. Snr_range1 and snr_range2 rarely increase. It is observed repeatedly under several adjusted power settings of GPS simulator.

On the attacker’s side, increasing the average signal strength of satellites is a natural strategy because the GPS receiver normally tries to capture the stronger signal. Furthermore, reducing the variability among the signal strengths of satellites can be a plausible way for GPS receiver to be locked onto at least one of them. Suppose that the variability is too high, one signal strength of the satellites can be high-powered at the same time. An excessively amplified signal has the possibility to be suspected as the spoofing signal. In this context, we believe that our selected parameters (i.e., mean, standard deviation, and difference) contain the distinguishable features for classifying the spoofing.

A DNN basically requires a large amount of training data and it often suffer from poor performance, also known as catastrophic forgetting, when it is exposed to very different environments. Thus, our model can adapt to similar types of the spoofer following the above-mentioned strategies, but it may not work well for totally different type of spoofing. Domain randomization [28] might be helpful, but it is still a challenging problem. Future work includes collecting the spoofing dataset generated by different types of spoofers and developing a versatile detection model for various attack scenarios.

### 4.3. Performance Comparison of Machine Learning Models

We performed a comparative test of 1D CNN (ResNet) and support vector machine (SVM) [29]. For the SVM, we used two types of kernels: linear and radial basis functions (RBF). The training/verification data ratio was 80:20. We used a test dataset collected from different regions of the training dataset. The authentic/spoofing signal ratio of the training dataset was 12:1. A number of training data is 33,056. The batch size is 128 and cross entropy is used for loss function. The test result of confusion matrix is given in Table 5. 

Precision, recall, and F-1 score were also considered to evaluate the performance of the learning models, as listed in Table 6.

All machine learning models were inferred accurately for authentic signals; however, they predicted different results for the spoofing signal. For the SVM, the RBF kernel performed better in terms of recall and F-1 score than the linear kernel. This experiment shows the 1D CNN (ResNet) outperforms the other machine learning models.

### 4.4. Measurement of Inference Time and Power Consumption

We evaluated the power consumption and inference time running on an embedded board to investigate real-time operability. The lower the energy consumption, the more advantageous the battery exhaustion. The shorter the inference time, the faster the operation of spoofing detection algorithm.

We adopted the NVIDIA Jetson AGX Xavier and Jetson Nano as the embedded boards for drone. First, we inspected the average power consumption for 5 min with respect to different operating modes (idle/running). In addition, we measured the average inference time of 30 tests when 1D CNN (ResNet) was executed on the board. The experimental test results are presented in Table 7.

As shown in Table 7, the Jetson AGX Xavier consumed more power; however, the inference time was shorter than that of the Jetson Nano. The average power of 8.26 W is consumed when Wi-Fi communication and GPS receivers are used simultaneously in a small UAV [30]. As the power consumptions of two embedded boards were less than 5 W, our algorithm is applicable for small UAVs. During drone flights, GPS data are normally acquired below 6 Hz. Because our average inference time was less than 50 ms, real-time operations are possible. In summary, our 1D CNN model is suitable for small UAV flights.

### 4.5. Field Tests and Results

#### 4.5.1. Validation Test for the GPS Spoofer

**Figure 8:**
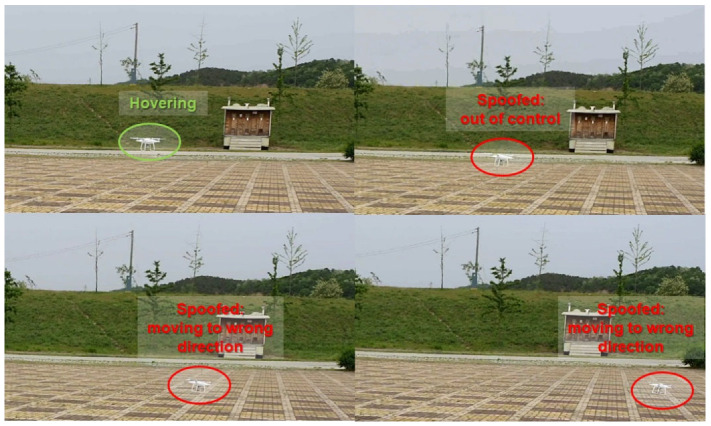
Test result of the Phantom drone.

To validate the performance of our spoofer, we selected the DJI Phantom 4, a representative consumer drone. The drone was exposed to a GPS spoofing attack in hovering mode. When a spoofing attack manipulated the drone, it became uncontrollable. Although position modulation occurred, the drone was unaware of it. The flight control system forced the drone to move to the predefined target position. However, the deviation from current position received by the GPS cannot be reduced by feedback control. Therefore, the flight control system fails to stabilize the drone, and it moves radically. As shown in Figure 8, the spoofing attack falsified the Phantom drone’s GPS receiver.

**Figure 9:**
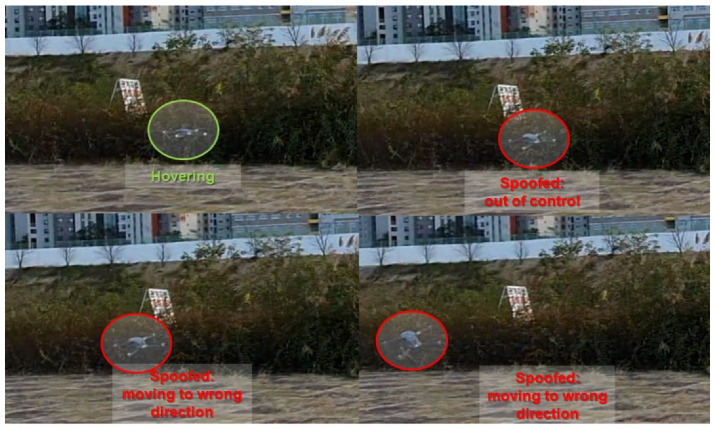
Test result of Mavic drone.

We also tested our spoofer’s performance for DJI Mavic drone. The drone was exposed to a spoofing attack again and we succeeded in deceiving the GPS receiver as shown in Figure 9. When most commercial drones encounter a problem in receiving GPS signals, the implemented fail-safe function is activated. To handle this emergency, they usually hover at the same location and try to find other GNSS services such as BeiDu, Galileo, and GLONASS.

As shown in Figure 8 and Figure 9, two commercial drones failed to find other GNSSs without having a chance to activate the fail-safe function. They immediately misconceived the intermediate spoofing signal as authentic signal.

#### 4.5.2. Flight Test Results

**Figure 10:**
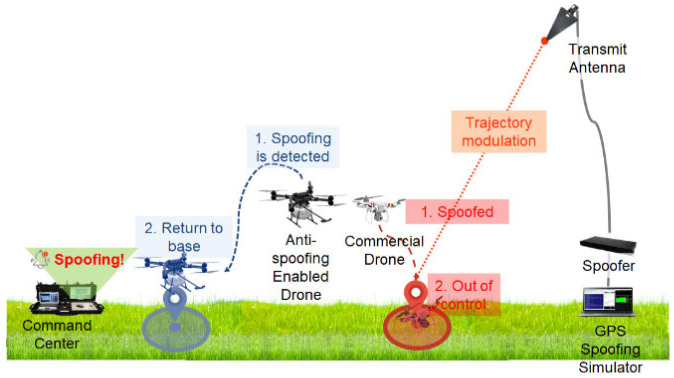
Spoofing test scenario.

**Figure 11:**
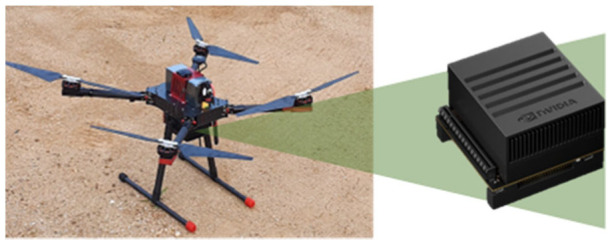
Anti-spoofing enabled drone and NVIDIA Jetson AGX Xavier.

We benchmarked our approach according to the scenario shown in Figure 10. Two drones were hovering in the field. One was our developed drone equipped with an anti-spoofing solution. We implemented the 1D CNN spoofing detection algorithm mounted on the Jetson Xavier (Figure 11). The other was a famous commercial drone, the DJI Phantom 4.

In our system, the command center sends a spoofing alarm when the algorithm interprets the received signal as spoofing signal. It immediately commands the drone to avoid spoofing. For instance, if the drone receives an alert signal (spoofed), it elevates its altitude and returns to the base.

However, without spoofing detection, the DJI Phantom failed to receive authentic GPS signals. Suddenly, the spoofed drone moved to an undesired position.

It is noted that the received SNR of drone will be much different between flight and hover state. It is affected by the flight speed, the relative direction to the spoofing antenna, and so on. Currently, our work is focused on reliably detecting the intermediate spoofing under a hover state.

**Figure 12:**
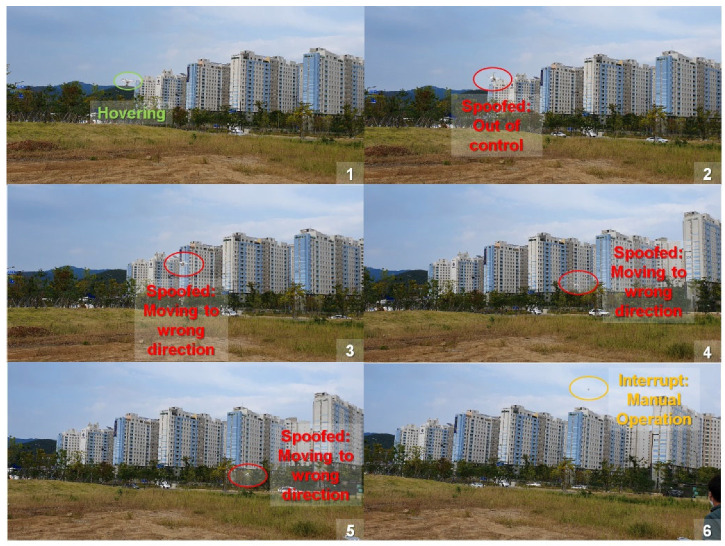
Spoofed DJI Phantom in the flight test.

In the flight test, we reused the spoofer whose efficiency was demonstrated in Section 4.5.1. We induced intermediate spoofing attacks on two drones in hovering status. Compared to our drone, the DJI Phantom reacted slowly to spoofing attacks. Notably, the response time can differ depending on GPS update frequency and flight control mechanism. After the Phantom drone received the spoofed signal, it recognized the deviation between the current and target positions. The flight control system forced the manipulated drone to drift to its previous position according to the hovering command. However, this deviation could not be reduced. Suddenly, it became unstable and out of control because of incorrect feedback. As shown in Figure 12, manipulated drone moved speedily in a wrong direction; thus, we had to interrupt the operation manually.

**Figure 13:**
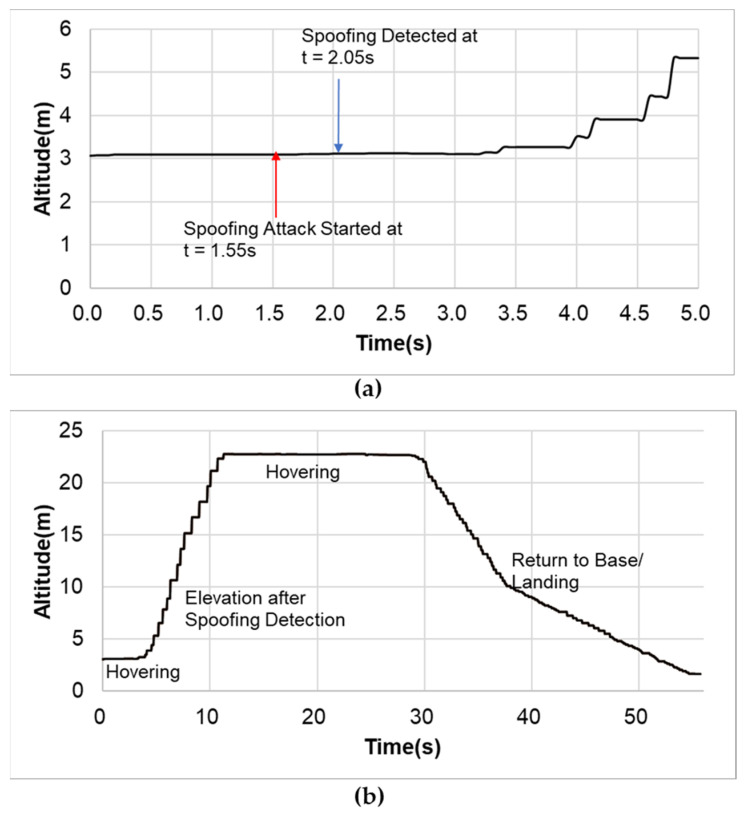
Flight trajectories; (**a**) from t = 0 to 5 s (**b**) during the flight test.

Our drone detected the spoofing attack within about 0.5 s as shown in Figure 13a. The spoofing detection and response time of ours is dependent of GPS signal receiving frequency, the drone’s processing and control mechanism, and the window slicing size as determined in Section 3.2. It detected the attack while maintaining the hovering mode. Once the spoofing alarm was ON, the drone moved up to approximately 20 m high to retreat from the hazardous area and safely returned home. The flight trajectories (time increment = 0.05 s) are given in Figure 13.

**Figure 14:**
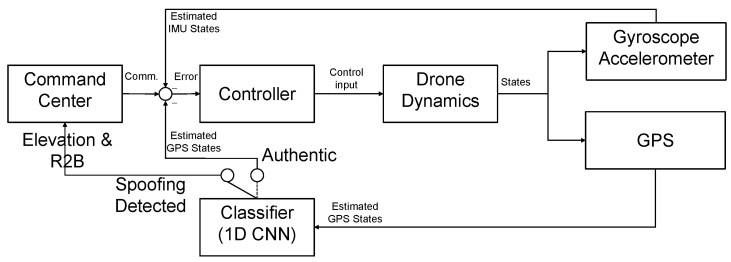
Feedback loop for the anti-spoofing enabled drone.

Figure 14 represents the feedback loop of an anti-spoofing enabled drone. The controller aims to minimize the error between the desired states and estimated states, including position, velocity, or attitude of the drone [31]. Monitoring the GPS states using 1D CNN classifier, we determine whether the current signal is authentic or spoofed in real-time. Once spoofing is detected, we cancel all the operations. Then, the return to base (R2B) command will be proceeded by moving to a higher altitude.

**Figure 15:**
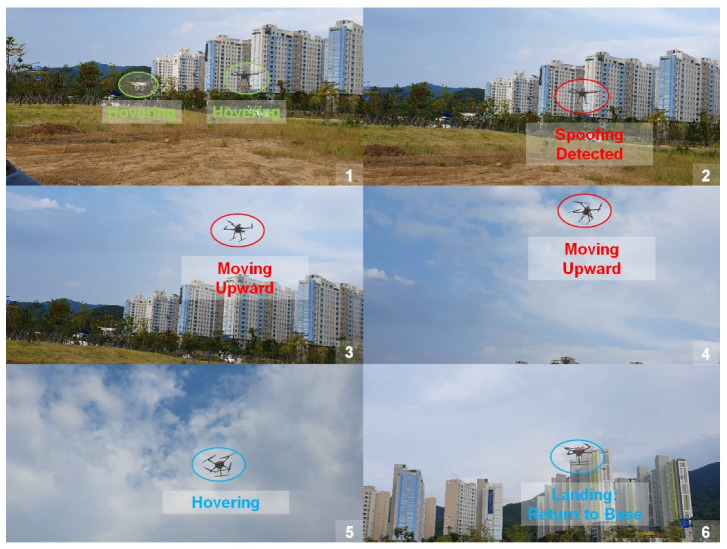
Spoofing detection and emergency landing of our drone in the flight test.

Following this feedback algorithm, our drone could detect the intermediate spoofing attack and perform emergency landing, as shown in Figure 15.

It showed fair detection performance in repeated experiments, however we found that its performance deteriorated, especially in multi-path environments (e.g., false alarms). Further works remain to improve the performance and reliability as follows:

- According to various spoofing attack scenarios, constructing database is essential. Sufficient data must be obtained under multi-path environments, various flight conditions and attacks by different types of spoofer.
- Ensemble techniques that combine several machine learning models can be adopted for better prediction. Accommodation of several features may also be helpful. They can compensate the weakness of different sensor data.
- Above approaches should be incorporated and well customized for operational safety over a period of time.

## 5. Conclusions

In this study, we investigated the GPS susceptibility of small UAVs and suggested that intermediate spoofing attacks would be an emerging threat to GPS-dependent platforms.

We have proposed a GPS spoofing detection method using 1D CNN to counter these attacks. We adopted the ResNet architecture, enabling us to detect most spoofed signals. Its performance in terms of precision, recall, and F-1 scores outperformed that of the SVM. Furthermore, the proposed method prevented position falsification before signal hijacking.

To validate the operability of small UAVs, we measured the power consumption and inference time on the embedded board. In the field test, our drone with the 1D CNN algorithm showed fair detectability of GPS spoofing attacks and successfully returned to the base.

Enhancing the performance and robustness of our model with respect to various environments, such as the flight conditions of drones and different spoofing attack scenarios, is our future research perspective.

## Figures and Tables

**Table 1:** Classification of GPS spoofing signal.

Signal Type	Meaconing	Generative Spoofing
Navigation message	same	deformed
GPS time Accompanied by jamming Target receiver mode	delayed o signal acquisition	synchronized x signal tracking

**Table 2:** Classification of GPS spoofing attacks.

Attack	Spoofer	Target Receiver Mode	Cost	Effectiveness	Practicality
Simplistic	GPS signal simulator	Signal acquisition	Low	Low	Low
Intermediate	Portable receiver spoofer	Signal acquisition/ tracking	Medium	High	High
Sophisticated	Multiple phase-locked Portable receiver-spoofers	Signal acquisition/ tracking	Very high	Very high	Low

**Table 3:** Type of NMEA-0183 interface.

Class	Data
TPV	position, time, velocity, error
SKY	prn, signal strength, azimuth, used, elevation

**Table 4:** Confusion matrix.

	Predicted Authentic Signal	Predicted Spoofing Signal
Actual Authentic Signal	True Positive (TP)	False Negative (FN)
Actual Spoofing Signal	False Positive (FP)	True Negative (TN)

**Table 5:** Confusion matrix of SVM and 1D CNN.

Model	SVM (Linear)		SVM (RBF)		1D CNN (ResNet)	
GPS Signal	Authentic	Spoofing	Authentic	Spoofing	Authentic	Spoofing
Authentic	8191	1	8189	3	8192	0
Spoof	54	22	14	62	2	74

**Table 6:** Performance comparison with machine learning models.

Model	SVM (Linear)		SVM (RBF)		1D CNN (ResNet)	
GPS Signal	Authentic	Spoofing	Authentic	Spoofing	Authentic	Spoofing
Precision	0.99	0.96	1.00	0.95	1.00	1.00
Recall	1.00	0.29	1.00	0.85	1.00	0.97
F-1 score	1.00	0.44	1.00	0.85	1.00	0.99

**Table 7:** Comparison of power consumption and inference time between Xavier and Nano.

Embedded Board	Mode	Power [Watt]	Time (ms)
Jetson AGX Xavier	Idle 30 W	2.52	-
	Running 30 W	4.65	28
	Running 15 W	4.25	32
Jetson Nano	Idle 10 W	1.42	-
	Running 10 W	3.63	30
	Running 5 W	2.75	47

## Data Availability

The data are not publicly available due to the policy of the Institute.
